# Cyst-Resembling Fibrous Dysplasia of the Mandible: A Case Report

**DOI:** 10.7759/cureus.106521

**Published:** 2026-04-06

**Authors:** Juyeon Cho

**Affiliations:** 1 Department of Dentistry, Keimyung University Donsan Hospital, Daegu, KOR

**Keywords:** cyst, diagnosis, fibrous dysplasia, mandible, maxilla

## Abstract

Fibrous dysplasia (FD) is a rare fibro-osseous condition usually occurring in the maxilla. FD lesions typically exhibit “ground-glass” radio-opacity, asymptomatic swelling, and bony expansion; however, lesions may manifest in different forms. A 29-year-old woman was referred with a cyst-suspected lesion located at the root apex of the mandibular premolar without expansion. An incisional biopsy was performed, which finally diagnosed the cyst-resembling lesion in the radiograph as FD. Consequently, unnecessary endodontic treatment of the related teeth was prevented. FD may occur with skin and endocrine features, termed McCune-Albright syndrome (MAS). Therefore, comprehensive and systemic screening for MAS should be performed when FD is encountered in dental clinics. Clinicians should recognize this less common presentation of FD in the clinical, radiographic, and histopathological fields to handle this diagnostic challenge.

## Introduction

Fibrous dysplasia (FD) is a benign fibro-osseous lesion characterized by the abnormal development of fibrous tissue or bone. FD is a disease of the skeletal stem cell in which both bone formation and resorption are affected [1]. It is prevalent in young adults in their 20s or 30s. Its etiology has been considered to arise from a functional mutation in GNAS1, which encodes a guanine nucleotide-binding protein that is involved in McCune Albright syndrome (MAS) [1]. FD can present as either a monostotic or polyostotic form, involving a single or multiple bones, respectively [1,2]. Approximately 70-80% of the disease manifests in a monostotic form in the long bones, while 20-30% manifests in a polyostotic form [1].

Asymptomatic swelling and bony expansion with cortical thinning are major features of FD [2]. A typical pathology is the replacement of the bone matrix with proliferating bone marrow cells. This phenomenon is responsible for the “ground-glass” radiolucency, which is a characteristic radiological feature of the disease [1]. However, the lesions may have variable presentations with age, thereby impeding proper diagnosis and management [3].

Herein, we report a case presenting with atypical clinical and radiological manifestations of FD in the mandible, resembling a periapical cystic lesion or fibro-osseous, cemento-osseous diseases.

## Case presentation

**Figure 1:**
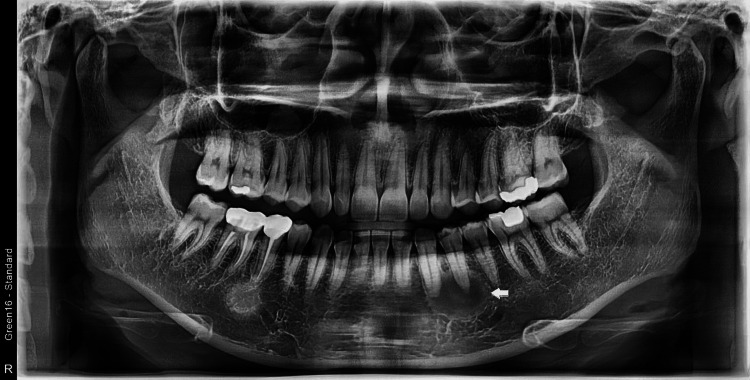
1. Dental panoramic radiograph A well-margined lesion with ground-glass appearance was found at the apices of the left mandibular canine and first premolar (arrow). The periapical lesion at the right mandibular second premolar was considered common, local osteosclerosis.

**Figure 2:**
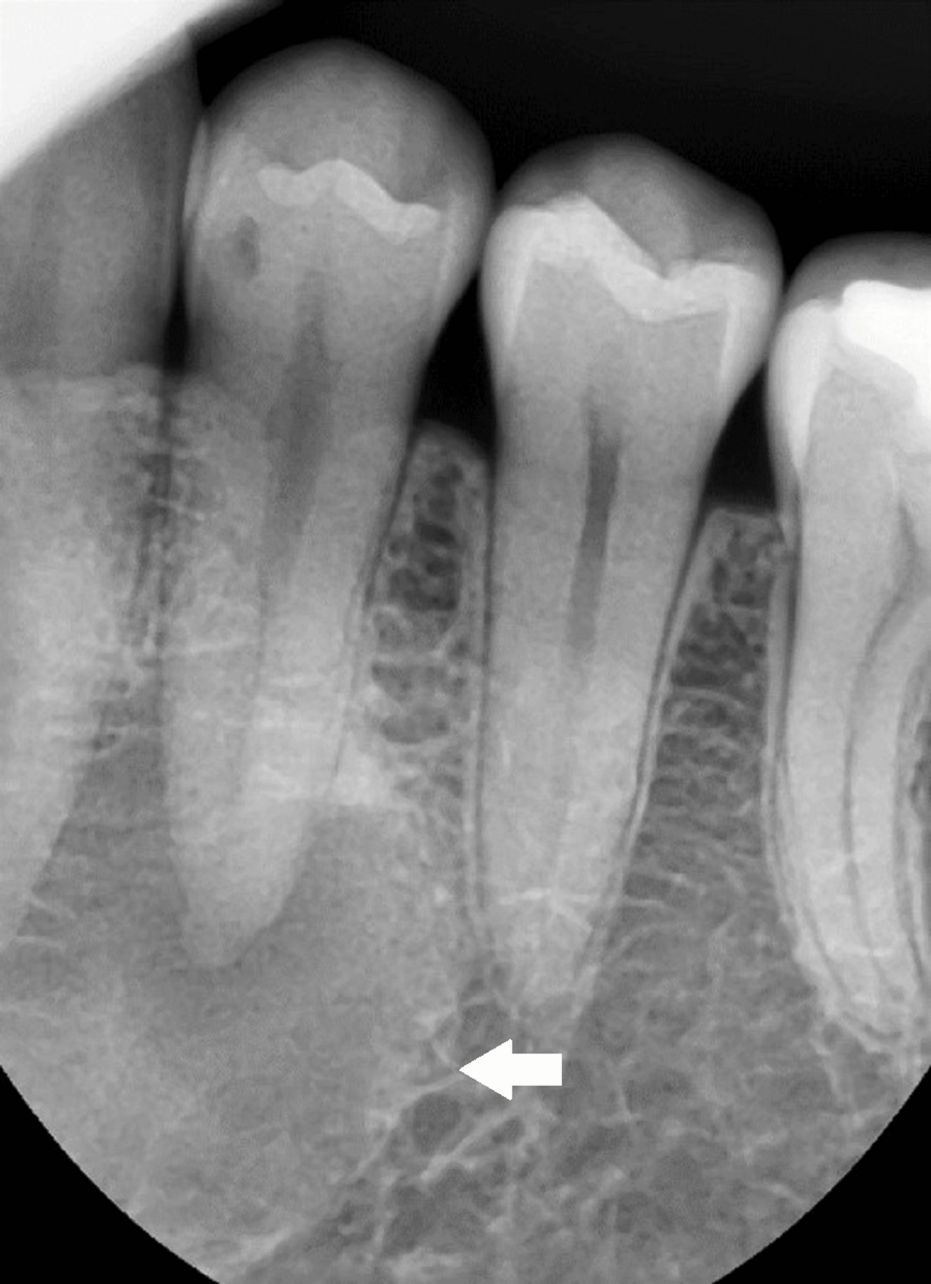
Periapical radiograph of the left mandibular first premolar and canine A cyst-resembling periapical lesion was found. The lamina dura of the first premolar and canine was not fully visualized, and sclerosis of the trabecular pattern is seen within the periapical lesion.

A 29-year-old woman was referred to our clinic for surgical enucleation of a cystic lesion in the left lower premolar. Intraoral examination results were normal, and no gingival swelling or definite bony expansion was observed. Extra-oral findings were also normal: the overlying skin was normal in color, and the patient presented no sensory abnormalities. Panoramic radiography revealed a well-delineated, round haziness around the roots of the left lower canine and first premolar (Figures 1, 2). The periapical lesion at the right mandibular second premolar was regarded as a common, local osteosclerosis. Therefore, the lesion on the contralateral side was not investigated at this time.

**Figure 3:**
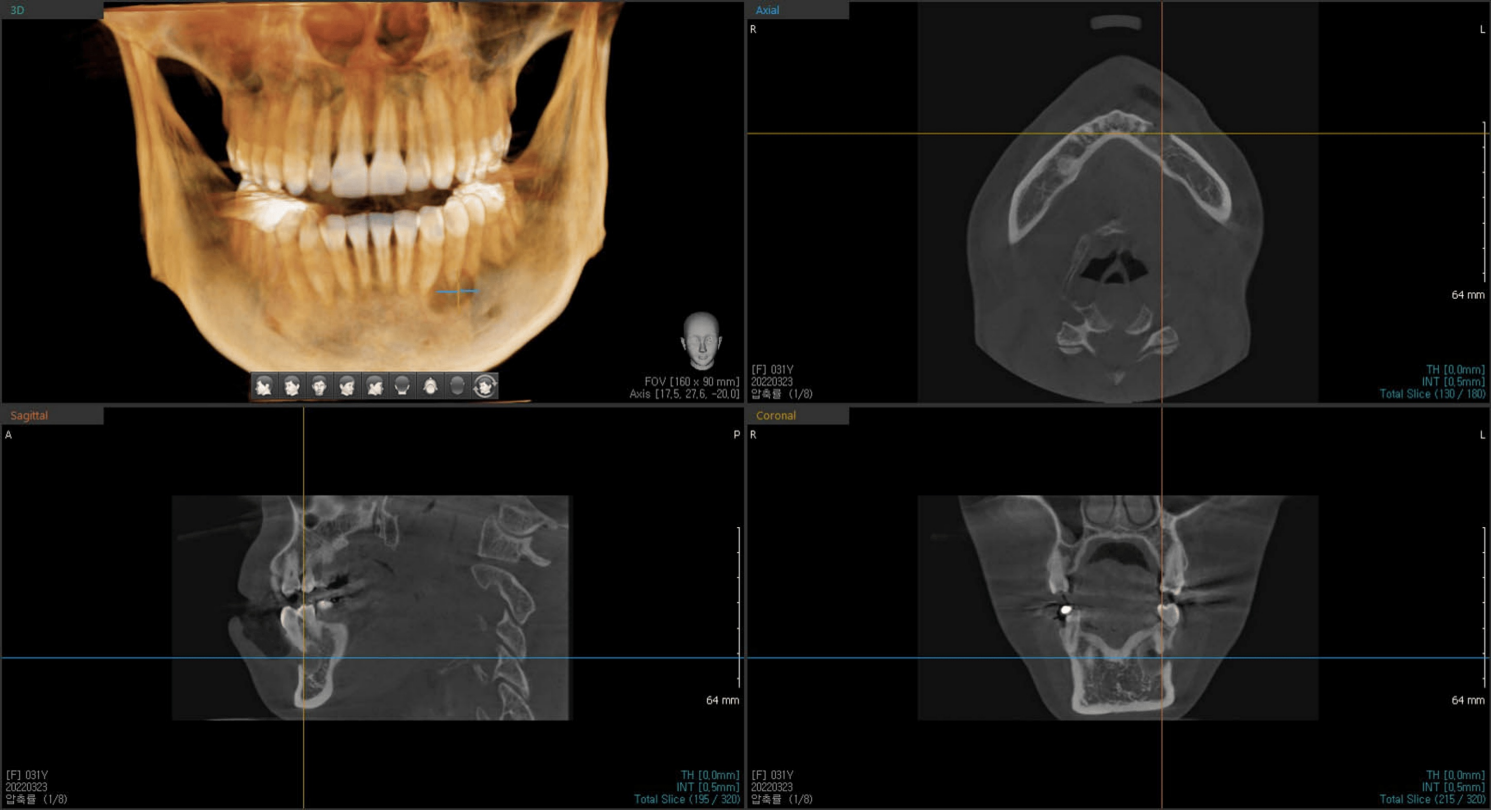
Dental cone beam computed tomography (CBCT) of the lesion 3D reconstruction view and multiplanar view of the lesion

**Figure 4:**
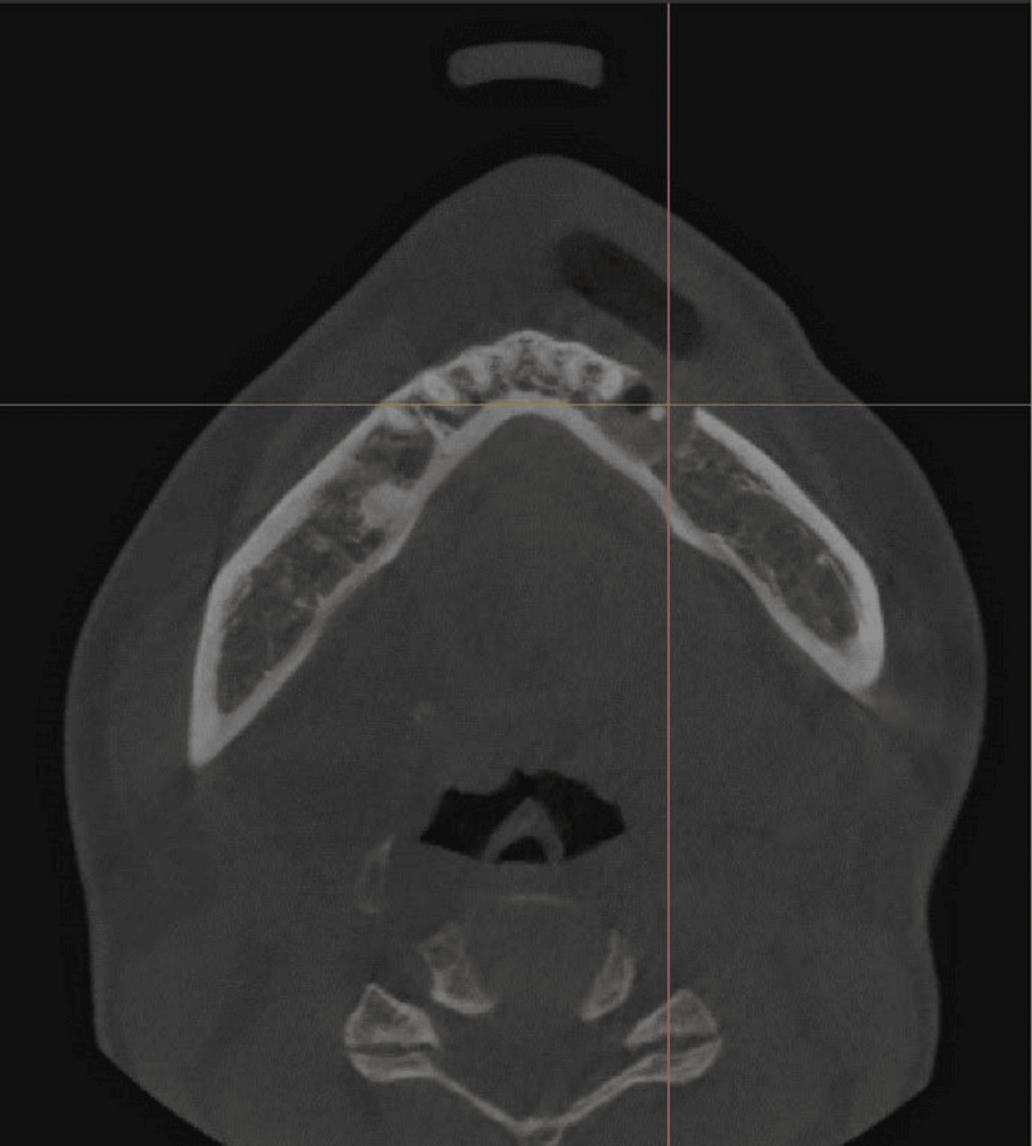
Dental cone beam computed tomography (CBCT) of the lesion A mixed heterogeneous hypodense lesion was noted in the axial view. This hypodense area represents regions where the tissue is less mineralized or where there is a preponderance of fibrous tissue compared to bone. No definite bony expansion was observed, but perforation of the buccal cortex existed.

**Figure 5:**
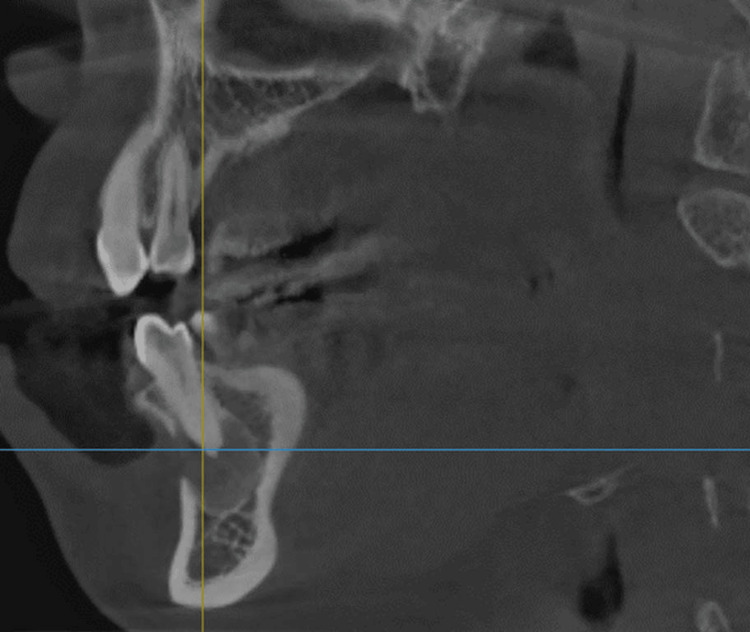
Dental cone beam computed tomography (CBCT) of the lesion A single, well-demarcated lesion with buccal cortical destruction was observed in the sagittal view.

Vitality tests of the affected teeth were positive for cold stimulus and an electronic pulp test, and were not sensitive to percussion. Probing depth and mobility test results were all within normal limits. Dental cone-beam computed tomography (CBCT) revealed a single, ill-defined, and diffuse-bounded lesion that thinned and perforated the buccal cortical bone. There was no clear, continuous cortical outline (sclerotic rim) that encapsulated the lesion. Instead, the lesion appeared to gradually blend into the adjacent healthy bone. The predominant internal characteristic was a heterogeneous "ground-glass" appearance. This describes a hazy, cloudy, or amorphous radiodensity that lacks the distinct trabecular pattern of normal cancellous bone. It is a mixture of more hyperdense (whiter) and hypodense (darker) areas, giving it a mottled or fibrillar texture. However, definite bony expansion was not observed around the lesion (Figures 3-5). The patient did not have a history of systemic disease or trauma.

**Figure 6:**
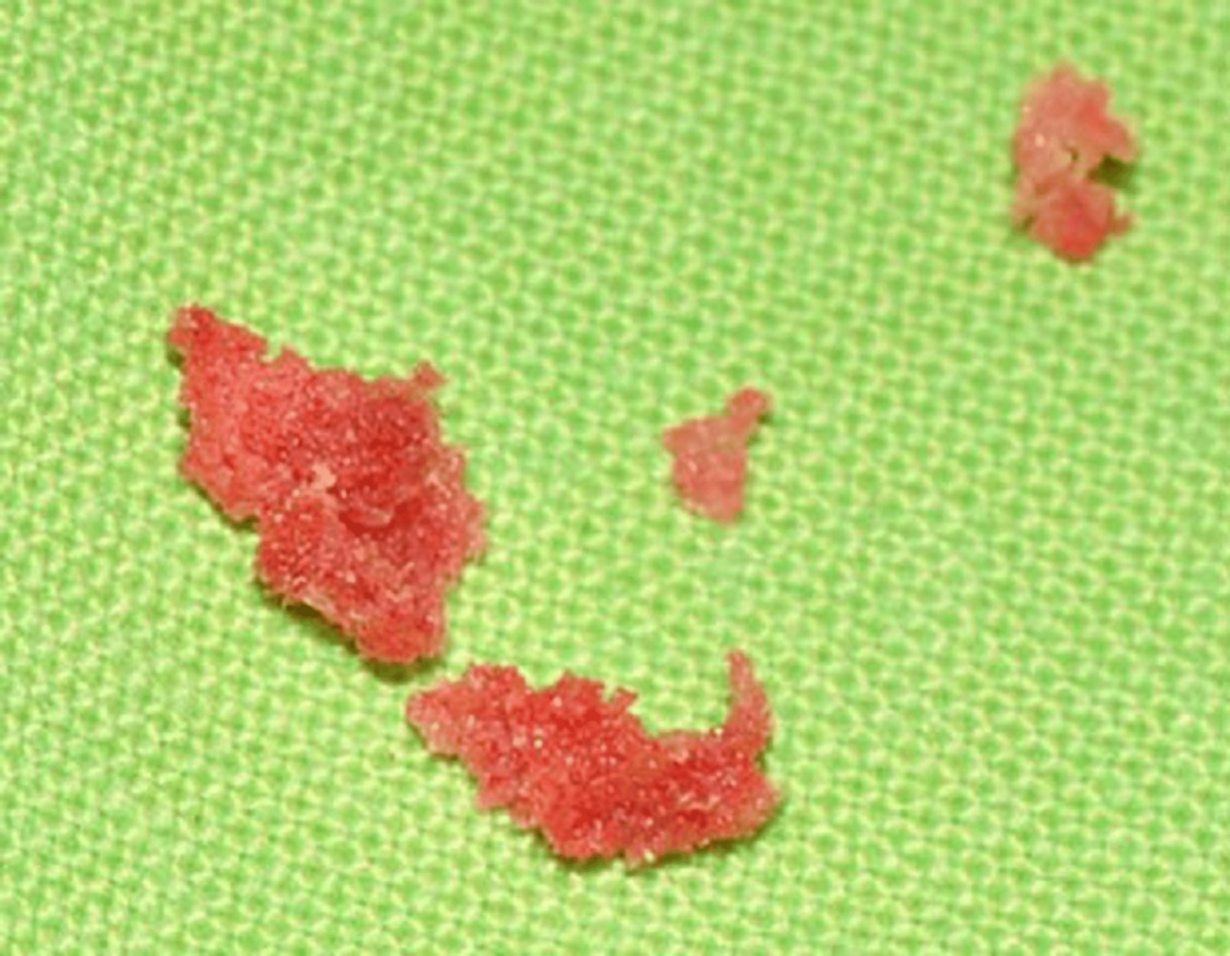
Gross specimen from the incisional biopsy Specimens indicated several sponge-like bony fragments.

An incisional biopsy was planned without root canal treatment of the affected teeth. Soft, sponge-like bone was observed during a biopsy (Figure 6). And the macroscopic sample showed a common appearance observed in FD.

**Figure 7:**
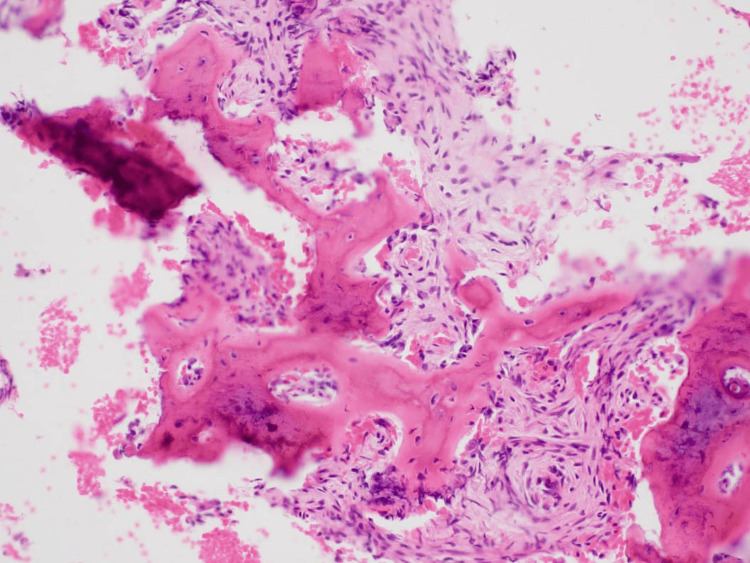
Hematoxylin-eosin staining of the biopsy sample Cell-rich fibrous stroma and irregular woven bony structures resembling a “Chinese character” (at original magnification ×200)

As the patient was planning a long trip abroad, whole-body scintigraphy for other bony involvement and other biochemical tests were not scheduled. Histopathological examination confirmed the diagnosis of FD by the presence of trabecular bone dispersed in the fibrous stroma. The tissue image showed a pink-stained fibrous stroma interspersed with irregular, immature bone trabeculae. These bone trabeculae typically lacked osteoblastic rimming, which was different from COD. Spindle cells were observed proliferating within the fibrous stroma. However, inflammatory cells, typically seen in inflammatory lesions, were not observed (Figure 7).

The patient was advised to undergo regular follow-up to monitor for any exacerbation or malignant transformations of the lesion, and provided informed consent for the publication of these findings.

## Discussion

FD is a benign bony lesion characterized by the replacement of normal bone with fibro-osseous tissue that exhibits various degrees of osseous metaplasia. FD is diagnosed based on clinical, radiographic, pathological, and molecular findings. For patients with monostotic FD, biopsy and identification of GNAS mutations may be required to confirm the diagnosis [1]. FD may present with skin and endocrine features, in which case it is termed MAS. Comprehensive screening for MAS should include bone scintigraphy to detect polyostotic lesions, for biochemistry, and to identify endocrine involvement [1,3]. In this case, only an incisional biopsy and histopathological examination were performed because the patient did not manifest any systemic or clinical symptoms. The differential diagnosis of FD is based on clinical, radiographic, and histological findings. Other fibro-osseous diseases (cemento-osseous dysplasia and ossifying fibroma), bone cysts, cementoma, and chronic osteomyelitis are classified as pathological conditions that mimic FD [4,5]. Also, desmoplastic ameloblastoma (DA), a relatively rare histological variant of ameloblastoma, commonly seen in the anterior region of jaws as a mixed radiopaque-radiolucent lesion resembling benign fibro-osseous lesions, should be considered as another differential diagnosis [6]. While the two diseases might look deceivingly similar in radiographs, histologically, DA is distinguished by small nests of compressed odontogenic epithelium embedded within a dense, collagenous stroma [6].

The lack of a well-corticated margin is a significant feature of FD, especially when differentiating it from other bone lesions like mature COD, which often presents with distinct, sometimes corticated, borders or a radiolucent halo, and tends to form more clearly defined masses. Histopathological features of COD exhibit fibrous connective tissue with small deposits of atypical cementum or bone tissue. As the lesion matures, the amount of cementum or bone tissue increases. Ossifying fibroma (OF) and cemento-ossifying fibroma (COF) are the lesions most frequently confused with FD. OF and COF present well-defined borders, whereas FD has a diffuse border and an expansive nature [5,7]. Radiographically, the “ground-glass” appearance of mixed radio-opacity/radiolucency may be characteristic of FD, in addition to cortical thinning and endosteal scalloping. These radiographic findings may vary according to age and manifestation location. Homogeneous ground-glass features are most common during childhood and adolescence, often becoming less radiolucent, more heterogeneous, and sclerotic with age [1]. The loss of the lamina dura of all the teeth within the lesion is another feature of FD; however, root resorption is uncommon [7]. The histopathological features of FD include irregularly shaped, lacking osteoblastic rimming woven bones that resemble “Chinese characters” dispersed in fibrous tissues [8]. This feature distinguishes FD from COD or Paget’s disease. Malignant transformation of FD is rare [9].

In our case, the lesion had a well-demarcated margin in both radiographs and CBCT images, which was an interesting and rare finding for FD. It did not exhibit bony expansion, as is usual for FD lesions. The round cyst or COD-like lesion with foggy radiodensity mimicked other cystic lesions filled with dense fluid or fibrous tissue. Additionally, the lamina dura at the apical thirds of both canine and premolar roots cannot be visualized. FD on CBCT scans mostly appears as a “ground-glass” homogenous hazy density and is grouped into predominantly cyst-like (11-22%), mixed (40-55%), and predominantly sclerotic (34-38%) types [7]. In this case, it resembled a cyst-like lesion, which is a rare presentation.

During the incisional biopsy of this case, the sample looked like a spongy, granular bone rather than granulomatous, cystic, or hard bone/cementum, which differentiates it from cystic lesions or COD. Early COD may appear soft or granulomatous due to the predominance of fibrous tissue, but it tends to be localized to specific areas rather than exhibiting the widespread and diffuse macroscopic characteristics of FD. Mature COD consists of very hard and dense cementum or bone tissue [4]. Therefore, during biopsy, it is often obtained as white, hard masses or fragments rather than reddish, granular pieces.

Most FD cases are of the monostotic type, with the maxilla more frequently affected than the mandible; furthermore, the monostotic type is mostly observed in young adults [10]. Conversely, this clinical case, occurring in the mandible, was diffuse-circumscribed, and the age of the patient was slightly higher than usual. The clinical and radiological findings confounded the diagnosis.

Surgical treatment for FD varies from biopsy to osteotomy [11]. Many surgical procedures tend to be postponed until skeletal maturity, which decreases regrowth and recurrence. In our case, only an incisional biopsy was performed owing to the lack of bony deformities and pain. Recurrence has been reported after limited bone curettage in FD [12]; therefore, the patient was informed of the refractory nature of the condition. Small monostotic lesions and those that are aggressive and/or refractory to contouring may require complete excision. However, the patient opted out of surgery and chose regular follow-up. Apart from surgical procedures, the use of bisphosphonates to decrease osteoclastic activity is regarded as a treatment option. Intravenous bisphosphonates appear to be valuable in FD-related bone pain; however, they should be used at lower doses to avoid the risk of osteonecrosis of the jaw [1,13].

The initial detection of FD/MAS may be possible by routine dental X-ray examination, although this may pose a significant diagnostic challenge. Clinicians should be aware of the main features of FD, as well as the significant variability in FD case presentations, to avoid misdiagnosis of periapical lesions and unnecessary endodontic treatment. Furthermore, treating FD using a team-based approach involving dentists, physicians, and surgeons is crucial. Long-term clinical follow-up is imperative, and the characteristic signs of FD must be thoroughly considered to appropriately refer patients for systemic workups if applicable.

## Conclusions

FD can mimic various periapical lesions such as cysts and other fibro-osseous diseases. The differential diagnosis of FD and related conditions like COD, OF, and COF is challenging both radiographically and histopathologically, but essential for the disease’s prognosis and subsequent surgical or conservative treatment planning. After FD is confirmed, the possibility of MAS should be considered, although it was not applicable in this case.

The present case highlights the importance of comprehensive clinical, radiographic imaging, like CBCT, and histopathological confirmation. The lesion appeared well-defined on the panoramic radiograph, as the outline could be clearly delineated from the surrounding bone. This good delineation in this current case was a departure from classic FD, where the lesion is usually with diffuse margins blending into surrounding bone. While the initial clinical impression by other dental providers was a periapical cyst, thorough investigation made the related teeth remain vital without unnecessary endodontic treatment. Early and appropriate diagnosis is essential to prevent unnecessary root canal treatment and to ensure long-term monitoring for potential recurrence or transformation.
